# Creepy cats and strange high houses: Support for configural processing in testing predictions of nine uncanny valley theories

**DOI:** 10.1167/jov.21.4.1

**Published:** 2021-04-01

**Authors:** Alexander Diel, Karl F. MacDorman

**Affiliations:** 1 School of Psychology, Cardiff University, Cardiff, United Kingdom; 2 Indiana University School of Informatics and Computing, Indianapolis, IN, USA

**Keywords:** anthropomorphism, configural processing, face perception, perceptual narrowing, Thatcher illusion, uncanny valley

## Abstract

In 1970, Masahiro Mori proposed the uncanny valley (UV), a region in a human-likeness continuum where an entity risks eliciting a cold, eerie, repellent feeling. Recent studies have shown that this feeling can be elicited by entities modeled not only on humans but also nonhuman animals. The perceptual and cognitive mechanisms underlying the UV effect are not well understood, although many theories have been proposed to explain them. To test the predictions of nine classes of theories, a within-subjects experiment was conducted with 136 participants. The theories’ predictions were compared with ratings of 10 classes of stimuli on eeriness and coldness indices. One type of theory, configural processing, predicted eight out of nine significant effects. Atypicality, in its extended form, in which the uncanny valley effect is amplified by the stimulus appearing more human, also predicted eight. Threat avoidance predicted seven; atypicality, perceptual mismatch, and mismatch+ predicted six; category+, novelty avoidance, mate selection, and psychopathy avoidance predicted five; and category uncertainty predicted three. Empathy's main prediction was not supported. Given that the number of significant effects predicted depends partly on our choice of hypotheses, a detailed consideration of each result is advised. We do, however, note the methodological value of examining many competing theories in the same experiment.

## Introduction

### The significance of the uncanny valley

**Figure 1.**
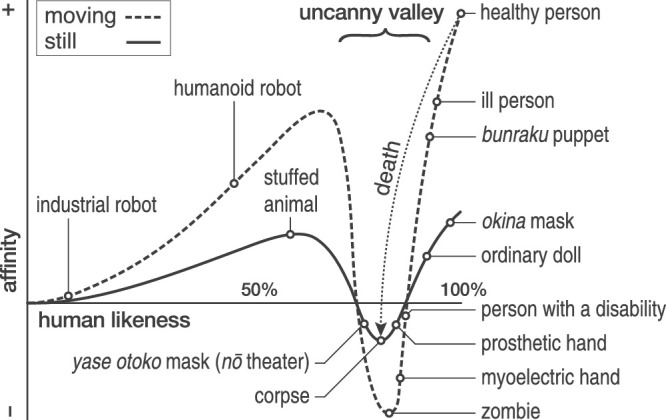
In 1970, Mori (2012) proposed an *N*-shaped relation between the degree of human likeness of an entity and the affinity it elicits in the observer. Affinity increases with human likeness up to a point before descending into a valley of eeriness only to rise out again as the entity becomes indistinguishable from a healthy person. In his graph, the valley is steeper when the entity is moving.

The use of human characters in computer animation, video games, and special effects has spurred growth in these markets, valued at more than $270 billion (Research and Markets, 2020). However, human characters have also been blamed for box-office flops and studio closures (Freedman, 2012). The effect identified as disrupting the appreciation of computer animation is the uncanny valley (UV). This term denotes an observer's negative affective reaction to human-looking entities, like android robots and computer-animated characters. The reaction manifests as a cold, eerie, repellant feeling. In 1970, Masahiro Mori proposed the UV effect, depicting it with a graph (Figure 1; Mori, 2012).

Since 2005, UV research has flourished, presenting a wide range of theories. Empirical studies have focused mainly on determining whether the UV effect exists or on testing the predictions of one of these theories (Kätsyri, Förger, Mäkäräinen, & Takala, 2015; Lay, Brace, Pike, & Pollick, 2016; Zhang et al., 2020). More than two-thirds of UV studies have found that the UV effect exists (Burleigh, Schoenherr, & Lacroix, 2013; Kim, Bruce, Brown, Visser, & Phillips, 2020; MacDorman & Ishiguro, 2006; Mathur & Reichling, 2015; Mathur et al., 2020; McDonnell, Breidt, & Bülthoff, 2012; Seyama & Nagayama, 2007). The effect has also been found in infants, children, and nonhuman primates (Brink, Gray, & Wellman, 2019; Lewkowicz & Ghazanfar, 2012; Siebert et al., 2020; Tinwell & Sloan, 2014; Steckenfinger & Ghazanfar, 2009). Nonetheless, its perceptual and cognitive mechanisms are not well understood, nor their neural basis (Rosenthal-von der Pütten, Krämer, Maderwald, Brand, & Grabenhorst, 2019; Saygin, Chaminade, Ishiguro, Driver, & Frith, 2012; Urgen, Kutas, & Saygin, 2018).

One way to evaluate theories is to compare the validity of their predictions experimentally across a range of stimulus conditions. Unfortunately, with only a few exceptions (e.g. MacDorman & Chattopadhyay, 2016, 2017; Mathur et al., 2020), emphasis has not been placed on theory comparison and falsification. Both are needed, given the large number of UV theories that have been proposed. To meet these needs, we have designed and carried out an experiment to test the predictions of nine classes of theories prevalent in the UV literature. This investigation is not exhaustive as we have omitted several theories (e.g. the autonomy–heteronomy distinction, expectation violation, mortality salience, and threat to human identity, as discussed in Ferrari, Paladino, & Jetten, 2016; Gahrn-Andersen, 2020; MacDorman, Ho, & Vasudevan, 2009b; MacDorman & Ishiguro, 2006; Ramey, 2005; Stein & Ohler, 2017; Urgen et al., 2018). It is also limited to still images, although some of the most compelling examples of the UV effect, even predating Mori's seminal essay, involve movement and more than one sensory modality (Jentsch, 1906; Thepsoonthorn, Ogawa, & Miyake, 2021).

A theoretical understanding of the uncanny valley will advance research in related areas, such as social perception, empathy, and human–computer interaction. It is also key to identifying design principles for creating android robots and computer-animated characters that avoid the uncanny valley.

### The methodology of uncanny valley research

UV research typically measures affective reactions to stimuli that vary in their degree of human likeness. Some studies instead represent the *x*-axis of Mori's graph as perceived realism (e.g. Schwind, Leicht, Jäger, Wolf, & Henze, 2018). Incremental transitions along the dimension are created by morphing (MacDorman & Ishiguro, 2006), editing (Mäkäräinen et al., 2014), or selecting stimuli (Mathur et al., 2020). Stimuli have been derived from photographs of humans, animals, robots, dolls, cartoon and computer-animated characters, and other sources. A few studies used only a small set of stimuli, for example, because the stimuli were difficult to obtain (e.g. physical androids) or because the study investigated the effects of manipulating a specific aspect like facial expressions (e.g. Brink et al., 2019; Palomäki et al., 2018; Tinwell, Nabi, & Charlton, 2013; Złotowski et al., 2015).

Mori labeled the *y*-axis of his graph *shinwakan,* a Japanese neologism indicating social presence and connection, which he translated into English as *affinity* (Mori, 2012). Affinity has been operationalized by one or more scales, typically Likert or semantic differential, designed to measure related constructs, such as *familiarity*, *likability*, *interpersonal warmth*, and reverse-scaled *eeriness* (Bartneck et al., 2009; Ho & MacDorman, 2010, 2017; Redstone, 2013).

One aim of UV research is to replicate the UV curve using stimuli that vary in human likeness (e.g. Kim et al., 2020, Mathur et al., 2020; McDonnell et al., 2012). Replicating the curve is not our current research goal. Another aim is to test differences in the UV effect between robots or computer-animated characters that vary in some aspect (e.g. Tinwell et al., 2013; Złotowski et al., 2015). Such experiments have already been conducted. Instead, this study aims to accomplish what no other has attempted: to evaluate more than two UV theories in a single experiment. Given the breadth of theories considered, the inquiry into each is limited. Follow-up experiments will be needed.

### Theories of the uncanny valley

Our review focuses on representative UV theories. We organized the theories into nine classes based on how their authors presented them; however, two broad divisions can be made: (1) theories defined in terms of an entity's features and their relations versus theories defined in terms of the entity as a whole; and (2) theories that apply generally versus theories that apply in a specific context (Table 1).

**Table 1.** Subdivisions of uncanny valley theories.

Context	Features and their relations	Entity as a whole
General	Configural processing	Category uncertainty
	Atypicality	Novelty avoidance
	Perceptual mismatch	
Specific	Mate selection	Threat avoidance
	Psychopathy avoidance	Empathy

The term *theory* has been used in various ways because the UV literature is interdisciplinary. For example, although the UV effect has been attributed to cognitive dissonance, this theory is not defined in terms of measurable properties of the stimulus but whether cognitions or actions are consistent. Thus, a degree of interpretation is required to test the predictions of certain theories in the context of vision science.

Configural processing theories predict that the UV effect is elicited by deviations in the configural pattern of familiar stimuli (Almaraz, 2017; Chattopadhyay & MacDorman, 2016; Kätsyri, 2018; Kätsyri, de Gelder, & Takala, 2019). Configural processing involves the holistic processing of a stimulus for rapid and accurate detection and differentiation (Kanwisher & Moscovitch, 2000; Rhodes, Brake, Taylor, & Tan, 1989). The development of configural processing depends on exemplars having invariant relations among their features, such as faces having two eyes, a nose, and a mouth set in specific relative positions (Pascalis et al., 2011). These first-order relations among features enable sensitization to second-order relations, such as whether the eyes are narrowly or widely set and to what extent (Collishaw & Hole, 2000; Rhodes et al., 1989).

Configural processing is impaired by stimulus inversion and other disruptions to the configural pattern. Stimulus inversion results in the stimulus being processed mainly from its features rather than from their second-order relations (Carbon et al., 2007; Kanwisher & Moscovitch, 2000; Maurer, Le Grand, & Mondloch, 2002).

Configural processing has been associated with a network of brain regions, including the fusiform face area for processing the face holistically, the occipital face area for processing facial features, and the superior temporal sulcus for processing facial dynamics (Haxby, Hoffman, & Gobbini, 2000). Faces elicit specific neurophysiological responses, including increased neural activity in the fusiform gyrus (Kanwisher, Dermott, & Chun, 1997) and a right-hemisphere negative event-related potential, the N170 (Eimer, 2000). Facial distortion increases the N170 response (Halit, de Haan, & Johnson, 2003), indicating increased processing as brain areas respond to anomalies (Olivares et al., 2015).

One role of configural processing in the UV effect has been established. Sensitivity to facial proportions increases with the realism of the face, and equally sized deviations from a face's original proportions are eerier in more realistic faces (Green, MacDorman, Ho, & Vasudevan, 2008; Seyama & Nagayama, 2007). Chattopadhyay and MacDorman (2016) proposed that perceptual narrowing causes small deviations from facial norms to appear unfamiliar and eerie (cf. Kelly et al., 2007; Lewkowicz & Ghazanfar, 2006; Pascalis, de Haan, & Nelson, 2002; Scott, Pascalis, & Nelson, 2007; Simpson, Varga, Frick, & Fragaszy, 2011). They found that inconsistency in feature realism made human and animal faces appear more unfamiliar and unreal, which elicited cold, eerie feelings.

Almaraz (2017) proposed that the UV effect is elicited by a mismatch between human-likeness cues from featural processing and those from configural processing. Unfortunately, Almaraz (2017) and Cheetham, Suter, and Jäncke (2014, experiment 3) used stimuli that lacked a valley in their transition from nonhuman to human, so they could not test whether nearly fully human faces elicited a UV effect when processed configurally. Kätsyri (2018) proposed that the perception of less-realistic faces activates configural processing less than real faces, thus making the visual system less responsive to structural deviations from norms.

Configural processing has also been found to develop through perceptual experience of non-face categories. For example, experts exhibit configural processing of stimuli related to their category of expertise (Carmel & Bentin, 2002; Diamond & Carey, 1986; Tarr & Gauthier, 2000; Vogelsang, Palmeri, & Busey, 2017). However, configural processing can also increase after only a few hours of being trained to individuate novel, nonface objects (Wong, Palmeri, & Gauthier, 2009).

The detection of deviations enabled by configural processing can produce disturbing effects, such as a grotesque appearance if only the eyes and mouth of a face are inverted. The grotesque appearance vanishes when the same face is then inverted. This effect is known as the Thatcher illusion. Observers generally cannot differentiate between an inverted Thatcher face and its inverted non-Thatcher counterpart (Donnelly et al., 2011; Thompson, 1980). Thus, inversion prevents a negative affective response to abnormal feature relations by disrupting configural processing (Bartlett & Searcy, 1993). The Thatcher effect has been found not only in the perception of infant and animal faces but also nonface objects like bicycles, cars, and strings of letters (Wong, Twedt, Sheinberg, & Gauthier, 2010). Uncanny faces may activate configural processing and thus heighten sensitivity to even subtle abnormalities in facial configurations. Deviations from human norms in these faces may then elicit an aversive response like the one elicited by the Thatcher illusion.

Atypicality theories predict that the UV effect is elicited by an exemplar that deviates strongly from the prototype of its category because of its unusual features. In its general form, atypicality theory does not specify the stimulus category or its characteristics (Kätsyri et al., 2015; Strait et al., 2017). However, in investigating atypicality in the perception of human face depictions, the strength of the UV effect has been found to increase with the depiction's realism (MacDorman, Green, Ho, & Koch, 2009a; Mäkäräinen, Kätsyri, & Takala, 2014). Thus, we propose an extended version of atypicality theory, atypicality*+*, which states that the UV effect is amplified by the stimulus's degree of human likeness.

Perceptual mismatch theories predict that the UV effect is elicited when different features of a single entity belong to different conceptual categories (e.g. a robot head paired with a human voice, Meah & Moore, 2014; Mitchell et al., 2011, human skin paired with enlarged eyes, Seyama and Nagayama, 2007, or disproportionate facial features or head and body, Rosenthal-von der Pütten et al., 2019). Moore (2012) proposed a Bayesian model of perceptual mismatch. He defined affinity for a stimulus as its probability of occurrence minus the individual's UV sensitivity times perceptual tension caused by the stimulus’ mismatched features.

Chattopadhyay and MacDorman (2016) revised Moore's model based on their empirical findings by replacing probability of occurrence with perceived familiarity. Their experiment found that the revised model applied to entities shaped like humans or other animals (zoomorphic) but not to entities shaped like plants or inanimate objects. Thus, as with atypicality and category uncertainty, the UV effect has been characterized as either independent of the stimulus category or dependent on the exemplar's degree of human likeness or perceived familiarity. We shall refer to the two different versions of perceptual mismatch models—the model applicable to any stimulus or only to zoomorphic entities—as mismatch and mismatch+, respectively.

Category uncertainty theories predict that the UV effect is caused by doubt regarding the category of an entity that appears ambiguous. In some versions, which we shall call category+, the effect is most strongly and reliably produced when the entity straddles one or more of the following mutually exclusive categories: living–nonliving, human–nonhuman, and real–unreal (Jentsch, 1906; MacDorman & Ishiguro, 2006; Ramey, 2005).

Broadly construed, category uncertainty theories include explanations of the UV based on balance theory (Tondu & Bardou, 2011), categorical perception (Burleigh et al., 2013; Cheetham, Pavlovic, Jordan, Suter, & Jäncke, 2013; Looser & Wheatley, 2010; Wang, Cheong, Dilks, & Rochat, 2020), categorization difficulty (Cheetham, Wu, Pauli, & Jäncke, 2015), categorization disfluency (Carr, Hofree, Sheldon, Saygin, & Winkielman, 2017; Seyama & Nagayama, 2009), category ambiguity (Burleigh & Schoenherr, 2015), category confusion aversion (Mathur et al., 2020), cognitive dissonance (MacDorman et al., 2009a), cognitive load (Yamada, Kawabe, & Ihaya, 2013), conflicting representations (Ferrey, Burleigh, & Fenske, 2015), and sorites paradoxes (Ramey, 2005). Despite the differences among these theories, they all imply that the UV effect correlates with categorization difficulty. Thus, category uncertainty theories can be tested collectively by comparing whether exemplars lying between two or more categories elicit eeriness more than those lying within a single category.

Categorization uncertainty theories face the following challenges. First, an uncanny entity could belong to an established category (e.g. a skeleton). Second, the eeriest stimuli are not always the hardest to categorize (MacDorman & Chattopadhyay, 2016; Mathur et al., 2020). Third, uncanniness is not identified as the experiential quality of the phenomenon under study in the literature of the theory adapted to explain the UV effect (e.g. cognitive dissonance, cognitive load, and perceptual dysfluency). Nevertheless, uncanny stimuli elicit a distinctive eerie experience (Mangan, 2015).

Novelty avoidance theories predict that the UV effect is elicited by stimuli that do not belong to an established category (Sasaki, Ihaya, & Yamada, 2017). One such theory states that categorizing an exemplar into a novel category produces an aversive response. The proposed purpose of this response is to avoid potential threats, such as unfamiliar people (Kawabe, Sasaki, Ihaya, & Yamada, 2017). Participants who self-rated higher on behavioral inhibition system scales also rated humanlike entities as eerier; this finding supports novelty avoidance theories (Sasaki, Ihaya, & Yamada, 2017). Novelty avoidance studies have been critiqued on theoretical, methodological, and empirical grounds (MacDorman & Chattopadhyay, 2016, 2017).

Novelty avoidance theories define novelty as not belonging to an established category. Therefore, if an uncanny stimulus belonged to an established category, such as the category “people with a disability” (Park, Faulkner, & Schaller, 2003), novelty avoidance theories would not explain this.

Mate selection theory predicts that the UV effect is elicited by visual cues of low fertility or low fitness in conspecifics, owing to an evolved perceptual-affective mechanism to prevent disadvantageous mating (Laue, 2017; MacDorman et al., 2009a; MacDorman & Ishiguro, 2006). This mechanism would have evolved to evaluate potential mates whose features elicit sexual arousal, namely *Homo sapiens* and other, now extinct, species of the same genus (e.g. *Homo neanderthalensis*). Because the mechanism is applied only to hominins, mate selection theory predicts that only humanlike entities elicit the UV effect. However, previous research has found that nonhuman animal stimuli also elicit the UV effect (Löffler, Dörrenbächer, & Hassenzahl, 2020; MacDorman & Chattopadhyay, 2017; Schwind et al., 2018; Yamada et al., 2013).

Psychopathy avoidance theory predicts that the UV effect is elicited by inauthentic emotions and other social cues that indicate psychopathic, hostile, or manipulative motives (Tinwell et al., 2013). As with mate selection theory, the mechanism is said to have evolved to evaluate other human beings, namely, those who could pose a threat; thus, if psychopathy avoidance theory holds, only human entities should elicit the UV effect.

Threat avoidance theories posit that the UV effect is an evolved perceptual-affective mechanism for avoiding danger. Specifically, pathogen avoidance theory predicts that the UV effect is elicited by an organism showing signs of infection (Burleigh et al., 2013; MacDorman & Ishiguro, 2006). Curtis, Aunger, and Rabie (2004) attributed threat avoidance to the disgust response. Sensitivity to disgust has been found to predict the UV effect (MacDorman & Entezari, 2015). Moosa and Ud-Dean (2010) proposed that the UV effect is caused by a more general mechanism for avoiding threats, as indicated by the presence of dead animals. Villacampa, Ingram, Corradi, and Olivera-La Rosa (2019) found a slight implicit association between creepy androids and moral disgust, which is an aversion to people who lack normal human motives (Rozin, Haidt, & McCauley, 2018).

All threat avoidance theories propose that the strength of the UV effect is proportional to an animal's morphological and behavioral similarity to a human being because similarity is a proxy for susceptibility to the threat owing to genetic relatedness (Ho, MacDorman, & Pramono, 2008; MacDorman et al., 2009a). Hence, threat avoidance theories imply only anthropomorphic and zoomorphic entities elicit the UV effect, not inanimate objects.

Empathy theories predict that the UV effect is elicited by empathy for an object known to be inanimate (e.g. Redstone, 2013). For example, the perceiver may automatically infer intentions from the object's actions while knowing the entity cannot think or feel. Gray and Wegner (2012) found that attributions of mind and experience to machines were associated with an eerie feeling, a finding reproduced by other researchers (Appel, Izydorczyk, Weber, Mara, & Lischetzke, 2020; Stein & Ohler, 2017). Misselhorn (2009) proposed that the repeated perceptual activation and cognitive inhibition of the concept human could provoke an uncanny feeling. This alternation may elicit free-floating anxiety if an observer perceives the negative experience enacted by an object, but cannot attribute the experience to the object itself because the object is inanimate (MacDorman & Entezari, 2015).

### Study aim

This study aims to test the predictions of nine classes of UV theories by drawing on their authors’ definitions. To this end, our experiment included stimuli not found in the literature, such as houses and greebles. These design choices were deliberate. Although the term UV is typically applied to three-dimensional objects designed to look human, including robots, computer models, and prostheses, we seek to test whether the predictions of the theories, as defined, hold for other stimuli they were meant to cover. This will help to determine whether the UV effect results from a general perceptual process or a specific one related to anthropomorphism or zoomorphism. If theory definitions were refined in light of our findings, that would contribute to the field.

### Hypotheses

We designed experimental conditions to test the predictions of the nine classes of UV theories introduced above. The following hypotheses characterize some of the predictions of the theories as we interpret them:

H1. Thatcher humans elicit a stronger UV effect than humans.
H2. Thatcher cats elicit a stronger UV effect than cats.
H3. Thatcher houses elicit a stronger UV effect than houses.
H4. Thatcherization elicits a stronger UV effect when applied to humans than to cats.
H5. Thatcherization elicits a stronger UV effect when applied to cats than to houses.
H6. Faces with distorted proportions elicit a stronger UV effect than undistorted faces.
H7. Greebles elicit a stronger UV effect than familiar objects like humans, cats, and houses.
H8. People with a disability resulting in facial dysmorphism elicit a stronger UV effect than people without one.
H9. Diseased body parts elicit a stronger UV effect than humans.

Configural processing theories predict that the configural processing of a misconfigured exemplar elicits the UV effect (H1–3, 6, 8, and 9) and that the strength of the effect is proportional to the extent of configural processing (H4 and H5). These theories do not predict a UV effect for novel objects (null for H7).

Atypicality theory predicts that deviations from the prototype of an existing category elicit the UV effect (H1–3, 6, 8, and 9). Atypicality, in its general form, operates irrespective of the category. Earlier, we proposed an extended form of atypicality theory, atypicality+, in which the UV effect is the combined effect of atypicality and human likeness (H4 and H5). Atypicality does not predict that exemplars belonging to a novel category elicit the UV effect; hence, greebles would not elicit the effect (null for H7).

Moore's (2012) perceptual mismatch theory predicts that inconsistencies among features, regardless of the stimulus category, elicit the UV effect (H1–3, 6, and 7). Mismatch+ theories additionally predict that the UV effect is the combined effect of feature inconsistency and human likeness (H4 and H5; e.g. MacDorman & Chattopadhyay, 2016). Greebles as novel objects do not have mismatched features (H8). People with a disability and diseased body parts have mismatched features (H9 and H10). However, they are not mismatched along the human–nonhuman or real–unreal dimensions described by mismatch+ theories (null for H9 and H10).

Category uncertainty theories predict that exemplars straddling category boundaries elicit the UV effect and that those lying within a category do not. A Thatcher human or human with distorted proportions could straddle the human–nonhuman boundary (H1 and H6). The same applies to Thatcher cats and houses (H2 and H3). Jentsch's formulation, category+, predicts a stronger UV effect on the human–nonhuman (H4) and living–inanimate (H5) boundaries. Novel objects are not predicted to produce a UV effect (null for H7).

Novelty avoidance theories predict that exemplars that do not belong to an established category elicit the UV effect (H1–3, 6, and 7), whereas exemplars that belong to an established category do not elicit the UV effect (null for H8 and H9).

Mate selection theory predicts a UV effect for human exemplars only (H1, 4, 6, 8, and 9). The theory does not predict a UV effect for Thatcher cats (H2), Thatcher houses (H3), and greebles (H7).

Psychopathy avoidance theory follows the same pattern as mate selection theory.

Threat avoidance theories predict a UV effect for humans (H1, 6, 8, and 9) and nonhuman animals (H2 and H5) with a stronger effect for humans than nonhuman animals (H4). It does not predict a UV effect for houses (H3) or novel objects (H7).

Empathy, a participant trait, was tested separately:

H10. The emotional quotient (EQ) predicts the UV effect.

Empathy theories predict that empathy for an inanimate object elicits the UV effect. An indirect consequence of this prediction is that the UV effect should be stronger in individuals with greater empathic abilities (H10).

## Methods

### Participant characteristics, sampling, and power analysis

Participants were recruited from Amazon Mechanical Turk. Inclusion criteria were at least fluent in English, no more than moderately impaired vision with correction, and passing the reverse-scaled items check (i.e. the items must correlate negatively with their unreversed counterpart).

Of 551 initial prospects, 136 participants met the inclusion criteria, consented, and completed the survey (61% men, *n* = 83). Participants ranged in age from 19 to 73 (median = 35, interquartile range = 29 to 48); 64.0% were White, 30.8% Asian, 9.6% Black or African American, and 5.9% Hispanic; 81.6% resided in the United States, 14.7% in India, 1.5% in Brazil, and 0.7% each in Italy, Mexico, and Pakistan.

In our previous study (MacDorman & Chattopadhyay, 2016), a 50% reduction in the realism of the whole face increased eeriness, *d* = 0.72, and a 50% mean reduction in realism of just the eyes and mouth increased eeriness, *d* = 0.26. For an effect size of 0.26, a 1-way repeated measures ANOVA with 10 conditions, 5 stimuli per condition, and 136 participants has a power of 0.90 (λ = 3.25, *df* = 1215.00).

The experiment was approved by Indiana University's Office of Research Administration (November 11, 2019, OHRP Category 7, Study No. 1910602465). Informed consent was obtained from all participants. Documentation of informed consent was waived under 45 Code of Federal Regulations (CFR) 46.117(c) or 21 CFR 56.109(c)(1). Human subjects research was performed under the provisions of the Declaration of Helsinki and complied with federal, state, and university standards, policies, and regulations.

### Research design

The experiment was a within-subjects design in which participants rated randomized stimuli from 10 stimulus conditions.

### Stimuli

Five randomly selected, standardized images comprised each of the following 10 stimulus conditions:

1. Humans: Human faces, two male and three female, shown face-on.
2. Thatcher humans: The same as condition 1, except with the eyes and mouth inverted.
3. Cats: Cat faces, shown face-on.
4. Thatcher cats: The same as condition 3, except with the eyes and mouth inverted.
5. Houses: The front of houses, shown squarely.
6. Thatcher houses: The same as condition 5, except with the front doors, at least two windows, and their trim inverted.
7. Distorted proportions: The same as condition 1, except with extremely distorted facial proportions.
8. People with a disability: Severe facial disfigurement, shown face-on.
9. Diseased body parts.
10. Greebles: Symmetric greebles.

**Figure 2.**
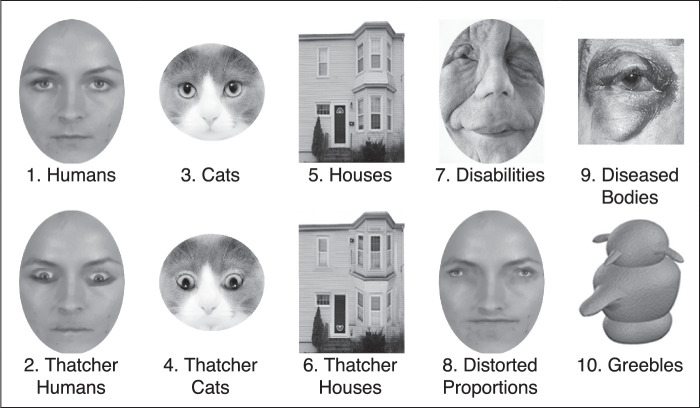
An image from each of the 10 stimulus conditions, retrieved from the database cited: 1. Humans (Chicago Face Database; Ma, Correll, & Wittenbrink, 2015); 2. Thatcher humans; 3. Cats (Cat Annotation Dataset; Zhang, Sun, & Tang, 2008); 4. Thatcher cats; 5. Houses (DalHouses database; Filliter, Glover, McMullen, Salmon, & Johnson, 2016); 6. Thatcher houses; 7. People with a disability (Shutterstock); 8. Distorted proportions; 9. Diseased body parts (Disgust-Related Images database; Haberkamp, Glombiewski, Schmidt, & Barke, 2016); 10. Greebles (CNBC Novel Objects database; Gauthier & Tarr, 1997).

A representative image from each condition is shown in Figure 2. For standardization, images were scaled to the same width and converted to grayscale; images of human and cat faces were cropped to exclude the ears, hair, and neck. Image artifacts were removed from Thatcher and distorted stimuli through smoothing. Image editing was performed with Adobe Photoshop CS6.

Five stimuli were used per condition. This design choice reflects a tradeoff between using more stimuli to mitigate confounds resulting from the lack of representativeness of a particular stimulus and using fewer stimuli to mitigate habituation, fatigue, and attrition.

### Scales and questionnaires

For the stimulus scales, the following nine items were adapted from the eeriness, warmth, and humanness indices for evaluating the UV effect (Ho & MacDorman, 2010, 2017): weird–dull, eerie–routine, creepy–bland, trustworthy–dishonest, friendly–hostile, warm-hearted–cold-hearted, human–nonhuman, and animate–nonliving. To better fit some of the stimuli, the following two scales were added: real–contrived and authentic–constructed.

These semantic differential scales were presented as visual analog scales, consisting of a horizontal bar with an adjective and its antonym on opposite ends (Funke & Reips, 2012; Reips & Funke, 2008). For each scale, the participant placed a mark on the bar. Its position was recorded as a decimal value between 0 and 100.0.

A standard 40-question adult EQ questionnaire was used to measure each participant's EQ (Baron-Cohen & Wheelwright, 2004). Representative items include “I really enjoy caring for other people,” “I can pick up quickly if someone says one thing but means another,” and “I find it easy to put myself in somebody else's shoes.” The Likert scales of the EQ questionnaire were converted into visual analog scales, as described above, with strongly agree and strongly disagree on opposite ends of the horizontal bar.

### Procedure

The experiment, implemented in Qualtrics as an online survey, was conducted from December 13 to 15 and 22 to 24, 2019. The participant determined the location and time of day.

After giving informed consent, each participant rated 50 images on the 10 scales listed above. Images were presented in random order. The participant then completed the EQ and demographics questionnaires. The experiment's average completion time was 50 minutes.

### Statistical analysis

Test statistics were interpreted at a 0.05 significance level. Pearson's *r* was interpreted with small = 0.1, medium = 0.3, and large = 0.5 thresholds. Cronbach's α was interpreted with acceptable = 0.7, good = 0.8, and excellent = 0.9 thresholds.

## Results

### Descriptive statistics and correlations

**Figure 3.**
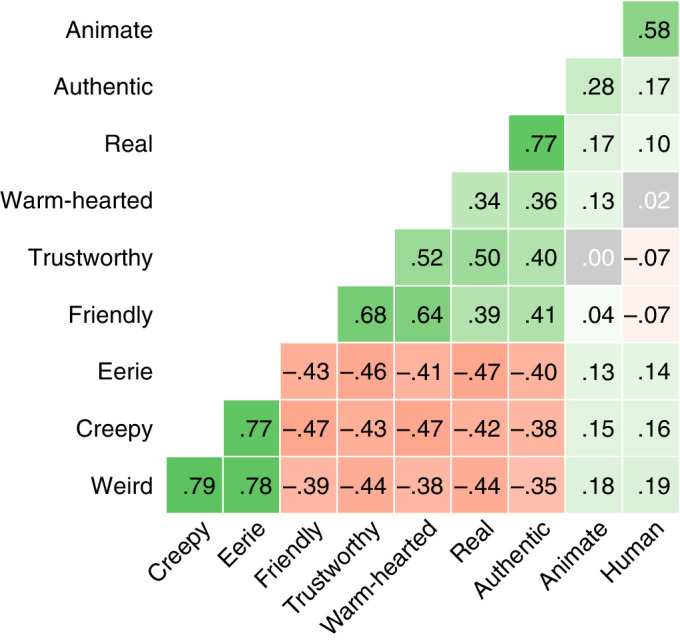
Pearson's correlation between each scale. All correlations were significant except those indicated by white text on a gray. *Note*: Only the positive semantic differential item is listed.

Table 2 lists the descriptive statistics for the UV scales. Figure 3 shows the correlations between their measurements.

**Table 2.** Descriptive statistics for the scales.

Item	*n*	*M*	*SD*	Skewness	Kurtosis
Weird	6800	55.37	34.40	–0.27	–1.35
Creepy	6800	53.97	34.20	–0.23	–1.36
Eerie	6800	51.61	34.30	–0.17	–1.39
Friendly	6800	50.42	26.29	0.02	–0.74
Trustworthy	6800	51.67	24.76	–0.03	–0.57
Warm-hearted	6800	48.26	26.61	0.13	–0.71
Real	6664	57.94	36.78	–0.32	–1.48
Authentic	6664	51.62	35.84	–0.11	–1.51
Animate	6800	58.60	40.97	–0.46	–1.52
Human	6800	44.21	43.84	0.20	–1.81

*Note*: Only the positive semantic differential item is listed.

### Factor analysis and index reliability

Exploratory factor analysis found the scales loaded on four factors (Table 3). The first and second factors were composed of the scales selected from the eeriness and warmth indices, respectively (Ho & MacDorman, 2010, 2017). The first factor was labeled eeriness. The second factor is analogous to affinity, the dependent variable in Mori's UV graph (see Figure 1). To make it easier to compare the first and second factors, the second factor was reverse scaled and labeled “coldness.” Items from the humanness index separated, loading on the third and fourth factors, labeled “realism” and “humanness,” respectively.

**Table 3.** Factor loadings of the scales in exploratory factor analysis.

Scale	Eeriness	Coldness	Realism	Humanness	Uniqueness
Weird–Dull	0.91				0.20
Creepy–Bland	0.87				0.21
Eerie–Routine	0.84				0.24
Friendly–Hostile		0.95			0.14
Trustworthy–Dishonest		0.61			0.43
Warm-hearted–Cold-hearted		0.61			0.48
Real–Contrived			1.01		0.00
Authentic–Constructed			0.64		0.37
Animate–Nonliving				0.89	0.19
Human–Nonhuman				0.64	0.58
Variance Explained	24.64	18.18	15.98	12.90	

*Note*: Minimum residual extraction method with oblimin rotation was used. Factor loadings < 0.4 are omitted.

Eeriness had excellent reliability, coldness and realism had good reliability, and humanness had acceptable reliability (Table 4). The reliability of eeriness was acceptable or good for all conditions, ranging from 0.77 (distorted proportions) to 0.89 (Thatcher houses). Coldness was acceptable or good for all conditions except greebles (α = 0.67). Real was acceptable or good for all conditions except houses (Spearman-Brown split-half reliability = 0.68). Humanness was unreliable for six conditions, including humans (highly leptokurtic and negatively skewed) and cats (sometimes rated animate and nonhuman). Eeriness was the only factor used in hypothesis testing.

**Table 4.** Psychometric properties of the scale indices.

DV	Items	*N*	*M*	*SD*	Reliab.	α_drop_	Skew	Kurtosis
Eeriness	3	6800	53.65	31.64	0.91^a^	0.87	–0.24	–1.17
Coldness	3	6800	49.88	22.29	0.83^a^	0.81	–0.10	–0.24
Realism	2	6664	54.78	34.15	0.87^b^		–0.21	–1.36
Humanness	2	6800	51.40	37.72	0.74^b^		–0.14	–1.47

*Note*: ^a^Cronbach's α; ^b^Spearman-Brown split-half; α_drop_ excludes the item with the lowest factor loading.

Eeriness was significantly correlated with coldness (*r* = 0.54, *p* < 0.001), realism (*r* = –0.47, *p* < 0.001), and humanness (*r* = 0.20, *p* < 0.001). Coldness was significantly correlated with realism (*r* = –0.49, *p* < 0.001) but not humanness (*r* < 0.01, *p* = 0.849). Realism was significantly correlated with humanness (*r* = 0.21, *p* < 0.001).

**Figure 4.**
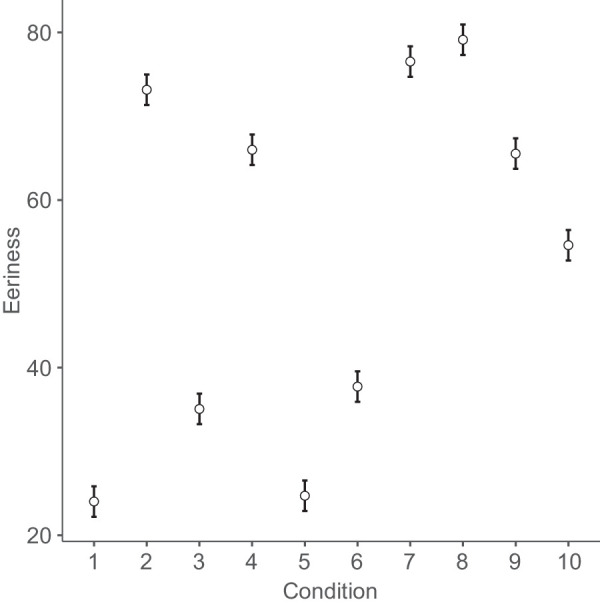
Mean eeriness and 95% confidence interval by condition: 1. humans, 2. Thatcher humans, 3. cats, 4. Thatcher cats, 5. houses, 6. Thatcher houses, 7. people with a disability, 8. distorted proportions, 9. diseased body parts, and 10. greebles.

Figures 4 ^5^ ^6^ to 7 plot mean eeriness, coldness, realism, and humanness by condition, respectively.

**Figure 5.**
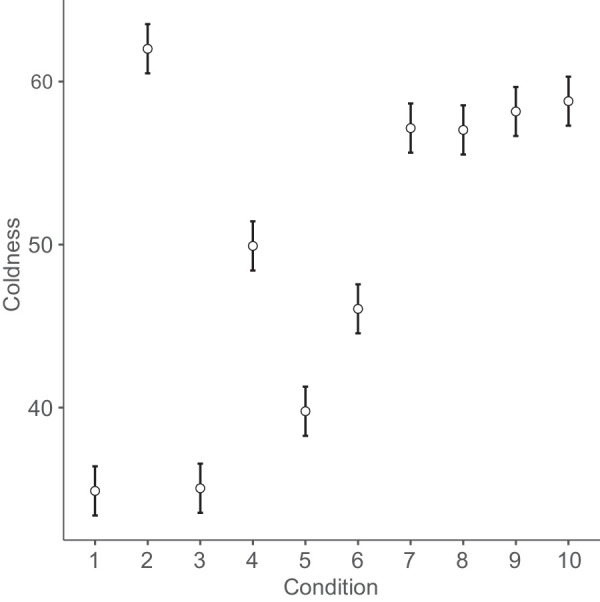
Mean coldness and 95% confidence interval by condition: 1. humans, 2. Thatcher humans, 3. cats, 4. Thatcher cats, 5. houses, 6. Thatcher houses, 7. people with a disability, 8. distorted proportions, 9. diseased body parts, and 10. greebles.

**Figure 6.**
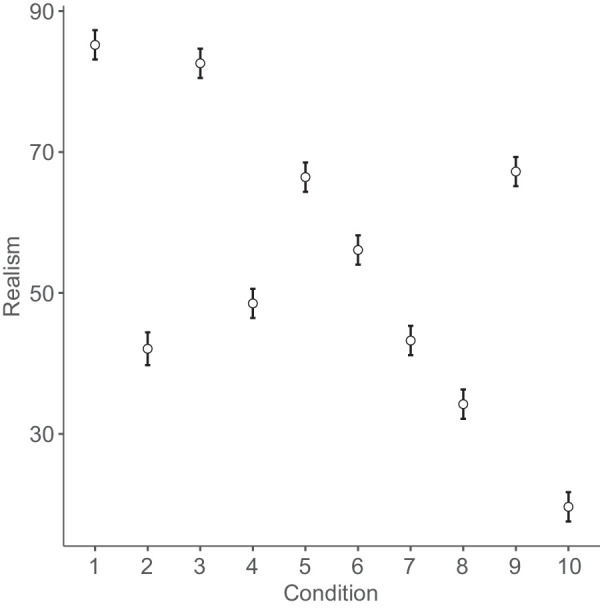
Mean realism and 95% confidence interval by condition: 1. humans, 2. Thatcher humans, 3. cats, 4. Thatcher cats, 5. houses, 6. Thatcher houses, 7. people with a disability, 8. distorted proportions, 9. diseased body parts, and 10. greebles.

**Figure 7.**
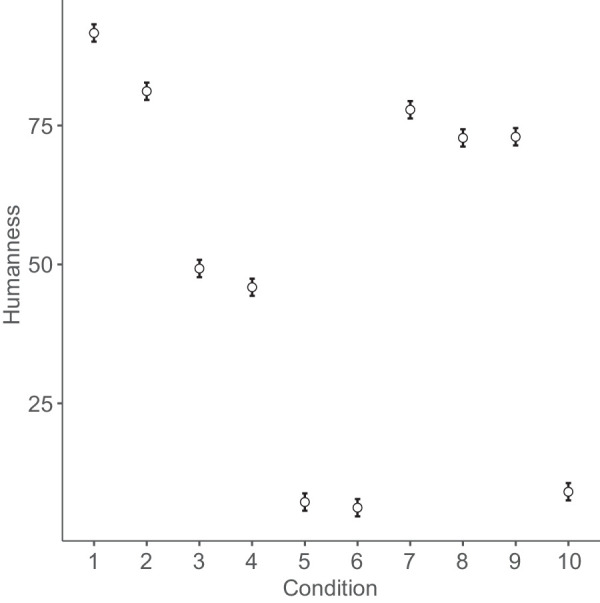
Mean humanness and 95% confidence interval by condition: 1. humans, 2. Thatcher humans, 3. cats, 4. Thatcher cats, 5. houses, 6. Thatcher houses, 7. people with a disability, 8. distorted proportions, 9. diseased body parts, and 10. greebles.

### Hypothesis testing: Eeriness

The design is within-subjects only. The eeriness factor was selected as the dependent variable because eeriness is considered the main indicator of the UV effect (Burleigh et al., 2013; Ho & MacDorman, 2010, 2017; Redstone, 2013).

Maximum likelihood estimation was used to fit a one-way linear mixed-effects model. Planned contrasts were used to compare the differences between the conditions.

All hypotheses were directional (i.e. condition *x* > condition *y*); therefore, the planned contrasts were one-tailed tests. Because some hypotheses describe nonorthogonal contrasts, the *p* values were adjusted for multiplicity. This correction was made by the Westfall method (Bretz, Hothorn, & Westfall, 2011). Condition had a significant effect on eeriness, *F*(9, 1215) = 225.16, *MSE* = 249.09, *p* < 0.001, $\eta_{p}^{2}$ = 0.63.

All hypotheses were supported (Table 5) except H10 (see below). Thatcher humans, cats, and houses were rated significantly eerier than normal humans, cats, and houses, respectively (H1–3). Thatcherization increased the eeriness of humans significantly more than cats (H4) and cats significantly more than houses (H5). Thus, the effect of Thatcherization increased with human likeness. (This pattern occurred, even though the proportion of the image that was inverted for human stimuli was less than for cat stimuli and still less than for house stimuli.) Human faces with distorted proportions were rated significantly eerier than undistorted faces (H6). Greebles as exemplars of novel objects were rated significantly eerier than normal humans, cats, and houses (H7). People with a disability were rated significantly eerier than people without one (H8). Diseased body parts were rated significantly eerier than humans (H9).

**Table 5.** Planned contrasts for eeriness and coldness.

	Eeriness				Coldness			
Hypothesis	*M*_diff_	*SE*_diff_	*t*	*p* value^*^	*M*_diff_	*SE*_diff_	*t*	*p* value^*^
H1. Thatcher human > human	49.13	2.03	24.26	< 0.001	27.12	1.63	16.63	< 0.001
H2. Thatcher cat > cat	30.93	2.03	15.27	< 0.001	14.86	1.63	9.11	< 0.001
H3. Thatcher house > house	13.02	2.03	6.43	< 0.001	6.28	1.63	3.85	< 0.001
H4. Contrast 1 > contrast 2	18.21	2.86	6.36	< 0.001	12.26	2.31	5.31	< 0.001
H5. Contrast 2 > contrast 3	17.90	2.86	6.25	< 0.001	8.59	2.31	3.72	< 0.001
H6. Distorted > human	55.10	2.03	27.21	< 0.001	22.14	1.63	13.58	< 0.001
H7. Novel > familiar	26.68	1.65	16.13	< 0.001	22.22	1.33	16.69	< 0.001
H8. Disability > human	52.50	2.03	25.93	< 0.001	22.26	1.63	13.65	< 0.001
H9. Diseased > human	41.51	2.03	20.50	< 0.001	23.27	1.63	14.27	< 0.001

*Note*: ^*^Westfall correction for multiplicity.

Empathy theories predict that the UV effect is elicited by empathy for an inanimate object. H10 states that the UV effect increases with empathetic abilities. However, a regression analysis revealed that an individual's EQ was a nonsignificant negative predictor of eeriness, *r* = –0.07, β = –0.07, *t*(678) = 3.68, *p* = 0.055, and explained a nonsignificant portion of the variance, *R*² = 0.01, adj. *R*² < 0.01, *F*(1, 678) = 29.75.

### Coldness

A one-way linear mixed-effects model revealed condition had a significant effect on coldness, *F*(9, 1215) = 80.12, *MSE* = 187.35, *p* < 0.001, $\eta_{p}^{2}$ = 0.37. Planned contrasts on coldness revealed the same results as on eeriness. Table 5 shows that greebles were significantly colder than familiar objects like normal humans, cats, and houses (H7).

A regression analysis revealed that an individual's EQ was a nonsignificant negative predictor of coldness, *r* = –0.04, β = –0.03, *t*(678) = –1.09, *p* = 0.274, and explained a nonsignificant portion of the variance, *R*² < 0.01, adj. *R*² < 0.01, *F*(1, 678) = 1.20.

### Theory evaluation

Table 6 indicates for each theory whether the effect stated in the corresponding hypothesis was predicted and whether it was found.

**Table 6.** Theory evaluation.

Hypothesis	Configural	Atypicality	Atypicality+	Mismatch	Mismatch+	Category	Category+	Novelty	Mate	Psychopathy	Threat
H1. Thatcher human > human	+	+	+	+	+	+	+	+	+	+	+
H2. Thatcher cat > cat	+	+	+	+	+	+	+	+	s	s	+
H3. Thatcher house > house	+	+	+	+	+	+	+	+	s	s	s
H4. Contrast 1 > contrast 2	+	s	+	s	+	s	+	s	+	+	+
H5. Contrast 2 > contrast 3	+	s	+	s	+	s	+	s	s	s	+
H6. Distorted > human	+	+	+	+	+	s	s	+	+	+	+
H7. Novel > familiar	s	s	s	s	s	s	s	+	s	s	s
H8. Disability > human	+	+	+	+	s	s	s	s	+	+	+
H9. Diseased > human	+	+	+	+	s	s	s	s	+	+	+
Predicted: sig. (+) : nonsig. (–)	8:0	6:0	8:0	6:0	6:0	3:0	5:0	5:0	5:0	5:0	7:0
Not predicted: sig. (s) : nonsig. (n)	0:1	0:3	0:1	0:3	0:3	0:6	0:4	0:4	0:4	0:4	0:2

*Note*: (+) The effect was predicted and found significant; (s) the effect was not predicted but found significant.

Configural processing theories predict that the configural processing of a misconfigured exemplar elicits the UV effect and that the effect's strength is proportional to the extent of configural processing. Thatcherization increased the eeriness of humans, cats, and houses (H1–3), as predicted, given that all three are processed configurally. Thatcherization also increased the eeriness of humans more than cats (H4) and cats more than houses (H5), as predicted, given that participants have greater exposure to humans than cats and that humans and cats have less variation in their configural pattern than houses. Faces with distorted proportions, either by artificial manipulation (H6) or because of disability or disease (H8), were also rated eerier, as predicted, as were diseased body parts (H9). However, configural processing failed to predict that novel objects like greebles would be rated eerier than familiar ones like humans, cats, and houses (H7), although greebles were still less eerie than five of the conditions.

Atypicality theories predict deviations from a category prototype elicit the UV effect, either irrespective of the category (atypicality) or proportional to its degree of human likeness (atypicality+). Six conditions deviated from a category prototype, and all six were eerier than their controls (H1–3, 6, 8, and 9). Atypicality+ additionally predicted that human likeness increases the effect of Thatcherization (H4 and H5). However, atypicality failed to predict that greebles would be eerier than familiar objects like humans, cats, and houses, because greebles as novel objects lack an established category prototype from which to deviate (H7).

Perceptual mismatch theories predict that inconsistencies among the features of an exemplar elicit the UV effect. Distorted proportions, which create second-order inconsistencies, increased eeriness as predicted (H6). Thatcherization, which creates inconsistencies between inverted and other features, also increased eeriness as predicted (H1–3). As predicted by mismatch+, human likeness increases the effects of Thatcherization (H4 and H5). However, mismatch+, which focuses on inconsistencies in such dimensions as human likeness and realism, failed to predict effects in perceiving people with a disability (H8) or diseased body parts (H9). Both groups are fully human and fully real. Mismatch, in its general form, predicted these effects. Mismatch and mismatch+ also failed to predict eeriness in novel objects (H7).

Category uncertainty theories predict that the UV effect is elicited by exemplars that straddle a category boundary. Even assuming Thatcher humans, cats, and houses straddled category boundaries, category uncertainty theories failed to predict five significant effects that atypicality+, configural processing, and threat avoidance predicted.

Novelty avoidance theories predict that exemplars not belonging to an established category elicit the UV effect. Greebles as novel objects were rated significantly eerier than familiar objects, a condition consisting of humans, cats, and houses (H7). However, even assuming Thatcherized and distorted exemplars were novel (H1–3 and 6), the theory failed to predict higher eeriness ratings of people with disabilities (H8) and diseased body parts (H9), although both should be established categories. Novelty avoidance also failed to predict the combined effect of Thatcherization and human likeness (H4 and H5).

Mate selection theory predicts that only humans elicit the UV effect because the underlying mechanism evolved to evaluate potential sexual targets. Although mate selection predicted higher eeriness ratings for all hypotheses involving human exemplars (H1, 4, 6, 8, and 9), it failed to predict those involving nonhuman exemplars: Thatcherization increased eeriness in cats and houses (H2 and H3) and increased it more in cats than houses (H5). It also failed to predict that novel objects would be eerier than familiar ones (H10).

Psychopathy avoidance theory makes the same predictions as mate selection theory regarding the hypotheses, with the results following the same pattern.

Threat avoidance theories predict that signs of contagious disease elicit the UV effect and exclude nonanimal stimuli as UV triggers. As predicted, diseased body parts were rated eerier than humans (H9). Assuming Thatcherization of humans and cats, distortion of humans, and disabilities were interpreted as signs of disease and greebles were interpreted as nonanimal, all other predicted effects were significant (H1, 2, 4–6, and 8). However, threat avoidance did not predict that Thatcher houses would be eerier than normal houses (H3) nor that novel objects would be eerier than familiar ones (H7).

Empathy theories predict that empathy for an inanimate object elicits the UV effect. However, the UV effect did not increase with the participant's empathetic abilities (H10).

### Data availability

Data analysis was performed in the *R* statistical computing environment (packages: jamovi, multcomp, nlme, performance, and psy). The dataset and *R* scripts for all analyses are available at https://doi.org/10.6084/m9.figshare.11888190.

## Discussion

### Evaluation of the tested theories

The experiment tested the predictions of nine different classes of UV theories. Configural processing and atypicality+ predicted eight out of nine significant effects; threat avoidance predicted seven; atypicality, perceptual mismatch, and mismatch+ predicted six; category+, novelty avoidance, mate selection, and psychopathy avoidance predicted five; and category uncertainty predicted three. Having fewer effects undermines the generality of a theory. It does not, however, falsify a theory because the same effect could have multiple causes, each explained by its corresponding theory. Empathy had a negative result for its key prediction, which could be investigated further by experimental methods.

Although the effects measured were too few to probe any one theory with sufficient thoroughness, they do identify predictions of the theories that need to be probed further. The implications of the experiment are examined below.

### Configural processing

Configural processing theories predicted eight out of the nine significant effects. Configural processing theories can also model underlying perceptual mechanisms, thus explaining the same observations predicted by atypicality and perceptual mismatch theories. Specifically, configural processing theories explain why sensitivity to atypical or mismatched features increases with exposure to the stimulus category: exposure increases the accuracy of judgments about second-order relations. Configural processing theories account for the amplifying effects of human likeness implicitly. The configural pattern of human faces is, through greater exposure, more firmly established than that of cats and houses. However, configural processing does not explain why novel objects should be eerier than familiar ones, although they were less eerie than five other conditions.

Future work on configural processing theories could first produce a UV along a human-likeness continuum and then examine how inversion flattens the valley (Almaraz, 2017). This approach could also help identify the relations among configural processing, the UV effect, and the stimulus category (e.g. real versus computer animated).

### Atypicality and perceptual mismatch

Atypicality and perceptual mismatch theories in their general form did not predict the increase in the UV effect with the stimulus's degree of human likeness. Atypicality*+* and mismatch*+* theories did.

There is evidence from the literature against these theories. Atypical and mismatched features sometimes elicit a more positive emotional and behavioral response than typical features. In biology, this phenomenon is exhibited by atypical features in supernormal stimuli: features that produce a positive response because they signal fitness may produce an even stronger positive response when exaggerated—sometimes to the point of impeding survival.

Supernormal stimuli are common in artistic depictions (Burch & Johnsen, 2019; Etcoff, Stock, Haley, Vickery, & House, 2011). They can be created with human faces through the use of cosmetics. Features with makeup appear less realistic than other features, which causes a mismatch in their feature realism. Nevertheless, makeup also increases female attractiveness significantly, and the size of this effect is large (Jones & Kramer, 2016). Similarly, slightly enlarging the eyes increases attractiveness, especially in female depictions (Baudouin & Tiberghien, 2004). Averaged faces, which are perceived as highly attractive, are also supernormal, being atypically symmetrical (Valentine, Darling, & Donnelly, 2004). Given that atypicality can produce positive reactions as well as negative ones, supernormal stimuli need to be examined in the context of the UV effect.

### Category uncertainty and novelty avoidance

Novelty avoidance failed to predict eeriness elicited by people with a disability, by diseases, and by the amplifying effect of human likeness on Thatcherization. Category uncertainty additionally failed to predict eeriness elicited by distortion or by novel objects. Thus, the proposed category-related effects were not necessary to elicit the UV effect. This result aligns with other experiments that have found that an exemplar need not straddle a category boundary to elicit the UV effect (MacDorman & Chattopadhyay, 2016; Mathur et al., 2020).

In novelty avoidance studies, stimuli typically morph by small increments from artificial to real human faces, thus spanning the human-likeness continuum. Their intermediate stimuli presumably also straddled the real–artificial boundary. Greebles satisfy neither of these conditions. However, Sasaki and colleagues (2017) define as novel a stimulus that cannot be categorized into an existing class. Greeble stimuli fit their definition (e.g. Gauthier & Tarr, 1997, used them as instances of a novel category).

Future research can test a broader range of stimuli fitting the given definition of novelty while controlling for other factors to evaluate novelty avoidance theories further. Future research can also test novelty avoidance by manipulating novelty experimentally, measuring the UV effect before and after participants are trained to categorize stimuli while controlling for habituation and familiarization.

### Mate selection, psychopathy avoidance, and threat avoidance

Threat avoidance theories exclude nonanimal stimuli as elicitors of the UV effect, and mate selection and psychopathy avoidance theories exclude nonhuman animal stimuli as well. Our results showed that nonhuman animal stimuli elicited a UV effect, reproducing past findings (Löffler et al., 2020; MacDorman & Chattopadhyay, 2016; Schwind et al., 2018; Yamada, Kawabe, & Ihaya, 2013). Moreover, our results also showed that nonanimal stimuli elicited a UV effect. This is a new finding, not found in studies including nonanimal objects as stimuli (e.g. MacDorman & Chattopadhyay, 2016). Thus, anthropomorphism and zoomorphism may not be necessary to elicit a UV effect, although they amplify it.

A fundamental question for any UV theory is why a stimulus should be experienced as uncanny. Had a cognitive mechanism evolved specifically to generate this aversive sensation or, as Mangan (2015) argues, is uncanniness a by-product of something else? The advent of realistic dolls, wax figures, computer animation, and android robots is fairly recent. If a need for the UV effect were rare in our evolutionary history, a perceptual or cognitive mechanism may not have evolved specifically to produce it. An uncanny entity may merely be eliciting and then violating neural expectancies about the human configuration. These failed expectancies would undoubtedly produce large feedback error signals (Friston, 2010; Rao & Ballard, 1999), which could manifest as an uncanny experience (Saygin et al., 2012; Urgen et al., 2018). Failed expectancies could then trigger orienting responses and, perhaps, an avoidance mechanism that had evolved for a different purpose.

### Empathy

Finally, empathy theories, like the one proposed by Misselhorn (2009), imply that a greater capacity for empathy should positively predict the UV effect. However, the results revealed that the capacity for empathy predicted eeriness negatively, although nonsignificantly. Perhaps greater empathy toward uncanny-looking entities mitigates negative affective evaluations of them. Thus, the relation between empathy and the UV could be the opposite of that previously proposed. Empathy-related theories based on attributions of mind or experience were not tested (Appel et al., 2020; Gray & Wegner, 2012).

### Limitations

Our stimulus conditions were mainly designed to evaluate lower-level visual and cognitive processing, not the higher-level processing of robots, computer-animated characters, and other complex dynamic objects. A more holistic consideration of how human–robot interaction contributes to the UV effect should include dimensions of social communication. These include timing, contingency, interactivity, and motion quality, and their relation to nonvisual modalities, such as speech and touch, not to mention verbal communication, interpersonal relationships, culture, age, and personality (Brink et al., 2019; MacDorman, 2019; MacDorman et al., 2009a, 2009b; Shin, Kim, & Biocca, 2019; Tu, Chien, & Yeh, 2020).

The novel objects condition used only greebles; this category may not be representative of novel objects in general. To ensure representativeness, this condition may require more varied exemplars. The relatively cold feelings felt for greebles may be attributable to their being computer renderings. Desaturating all images of color to make familiar objects more comparable to the monochromatic greebles may have reduced their ecological validity.

The diseased body parts condition lacked an adequate control condition, such as the same body part without disease. A better approach, given that human faces were used as controls for other conditions, would be to use similarly photographed diseased faces.

Turning to methodology, there is a degree of arbitrariness in evaluating classes of theories by the relative number of significant effects predicted. That number depends on the particular list of hypotheses and set of stimulus conditions selected.

The eeriness and coldness indices were reliable for all 10 stimulus conditions. Although they gave identical results for the tested hypotheses, their factor analysis, reliability coefficients, means by condition, and correlations indicated they measured different constructs. If combined, their reliability would fall to 0.19. However, items from the humanness index, which had loaded on one factor in robot and computer animation studies (Ho & MacDorman, 2010, 2017), separated into two factors, realism and humanness, which were not reliable in all conditions. Because neither was a dependent variable, this limitation does not affect the hypotheses.

The mechanisms underlying aversion to Thatcher houses may differ from those underlying aversion to androids or computers with feelings. Depending on the situation, these phenomena could have different perceptual, cognitive, and affective mechanisms. Moreover, the mechanisms underlying, for example, configural processing and threat avoidance could operate in parallel. If so, more than one theory may be required to explain the UV effect (Gahrn-Andersen, 2020; Mangan, 2015; Wang, Lilienfeld, & Rochat, 2015). Different theories about the same mechanism may complement each other by focusing on different levels of description: neural, perceptual, cognitive, behavioral, evolutionary, and so on.

## Conclusion

This experiment tested the predictions of nine widely varying classes of UV theories. Configural processing and atypicality+ theories had the greatest number of predictions with significant effects.

For all theories, except novelty avoidance, the experiment used the same stimulus conditions. This approach is new. Past experiments have simultaneously tested the predictions of one or, at most, two theories.

Although the conditions were selected based on the predictions of each type of theory, the experiment only partially tested their assumptions. Future research should investigate the theories in more detail to explain the UV's causes and mechanisms, which in turn should help designers avoid it.
